# Increased Number of Neurons in the Cervical Spinal Cord of Aged Female Rats

**DOI:** 10.1371/journal.pone.0022537

**Published:** 2011-07-20

**Authors:** Enrique L. Portiansky, Fabian Nishida, Claudio G. Barbeito, Eduardo J. Gimeno, Rodolfo G. Goya

**Affiliations:** 1 Laboratorio de Análisis de Imágenes, School of Veterinary Sciences, National University of La Plata (UNLP), La Plata, Argentina; 2 INIBIOLP-Histology "B", School of Medicine, National University of La Plata (UNLP), La Plata, Argentina; University of Michigan School of Medicine, United States of America

## Abstract

In the brain, specific signaling pathways localized in highly organized regions called niches allow the persistence of a pool of stem and progenitor cells that generate new neurons in adulthood. Much less is known about the spinal cord where a sustained adult neurogenesis is not observed. Moreover, there is scarce information concerning cell proliferation in the adult mammalian spinal cord and virtually none in aging animals or humans. We performed a comparative morphometric and immunofluorescence study of the entire cervical region (C1-C8) in young (5 mo.) and aged (30 mo.) female rats. Serum prolactin (PRL), a neurogenic hormone, was also measured. Gross anatomy showed a significant age-related increase in size of all of the cervical segments. Morphometric analysis of cresyl violet stained segments also showed a significant increase in the area occupied by the gray matter of some cervical segments of aged rats. The most interesting finding was that both the total area occupied by neurons and the number of neurons increased significantly with age, the latter increase ranging from 16% (C6) to 34% (C2). Taking the total number of cervical neurons the age-related increase ranged from 19% (C6) to 51% (C3), C3 being the segment that grew most in length in the aged animals. Some bromodeoxyuridine positive-neuron specific enolase negative (BrdU^+^-NSE^−^) cells were observed and, occasionally, double positive (BrdU^+^-NSE^+^) cells were detected in some cervical segments of both young and aged rats groups. As expected, serum PRL increased markedly with age. We propose that in the cervical spinal cord of female rats, both maturation of pre-existing neuroblasts and/or possible neurogenesis occur during the entire life span, in a process in which PRL may play a role.

## Introduction

The discovery that neurogenesis occurs in the brain of adult human and of nonhuman primates has generated a great deal of interest [1], [2]. Indeed, the possibility that the adult central nervous system (CNS) retains the potential for neurogenesis opens the prospect for new interventive therapies aimed at stimulating the genesis of specific neurons (e.g., dopaminergic nigral neurons) in patients affected by neurodegenerative diseases and other disorders of the adult/aged CNS [3], [4].

Although sustained neurogenesis has been reported in the adult rat brain [5], [6], it was not detected in the spinal cord of intact adult male rats [3]. The existence of neurogenesis has been explored neither in the spinal cord of female nor in older (>4 mo.) male rats. In fact, there is scarce information even on the general morphological changes in the spinal cord of aging rats. In previous studies we have observed that there is an increase in the number of neurofilaments present in the gray matter of aged rats [7], changes in the lectinhistochemical pattern [8], a complete loss of neuron-specific nuclear protein (NeuN) immunoreactivity in cervical, thoracic and lumbar segments of aged female rats [9], as well as a decrease in the expression of a phosphatase and tensin homologue on chromosome 10 (PTEN), a tumor suppressor gene known to play an important role in the regulation of cell size [10].

In neither case the observed changes were due to inflammatory or other pathological conditions since the number of glial cells did not increase [7], [11].

As part of a systematic characterization of morphological age changes in the brain and spinal cord of female rats, we morphometricaly and immunohistochemicaly assessed the cervical segments of aged female rats and compared them with the same segment of young counterparts. We report here that besides the previously reported age changes in female rats described above, aging is also associated with an increase in the number of neurons in the cervical spinal cord. Since prolactin (PRL) has been reported to induce neurogenesis in the forebrain of adult female mice [12], [13] we also measured serum levels of PRL in our female rats and found a significant increase with aging.

## Methods

### Animals and specimen collection and processing

Young (4–5 mo.) (n = 7) and aged (30 mo.) (n = 7) female Sprague-Dawley rats, raised in our aging rat colony, were used. The young females were virgin while the aged animals were retired breeders. Animals were housed in a temperature-controlled room (22±2°C) on a 14∶10 h light/dark cycle. Food and water were available *ad libitum*. In our rat colony, the average 50% survival time for females, studied in groups of 50–60 animals, is 30 mo. Around 10–12 mo. of age, reproductive cessation occurs in our females. All experiments with animals were performed according to the recommendations in the Guide for the Care and Use of Laboratory Animals of the National Institutes of Health. The protocol was approved by the Committee on the Ethics of Animal Experiments of INIBIOLP's Animal Welfare Assurance No A5647-01.

Euthanasia was performed according to the Guidelines on the Use of Animals in Neuroscience Research (the Society of Neuroscience) and the Research Laboratory Design Policy and Guidelines of NIH. Immediately before sacrifice rats were placed under general anesthesia by injection of ketamine hydrochloride (40 mg/kg, i.p.) plus xylazine (8 mg/kg; i.m.) and blood samples were taken from the tail veins. The corresponding serum was stored at −20°C until hormone assay.

After blood sampling, the animals were intracardiacally perfused with a buffered saline-paraformaldehyde 4% solution during approximately 30–45 min. The head of the rats was cut 1–2 mm rostral to the occipito-atloideal junction using an electric rotary saw. The vertebral column was then removed and posfixed in 10% buffered formaldehyde for 24 hs. The spinal cord was then dissected, immersed in cryopreservation buffer (sucrose 30%; polyvinylpyrrolidone 1%; etilenglycol 30% phosphate buffer 1M 1%; DW to 100 ml) and stored at −20°C until use.

Coronal sections of cervical segments were performed under a magnifying glass. Because the spinal cord segment is, by definition, that part of the cord which gives rise to those root fibers that unite to form a pair of spinal nerves [14], the caudal border of a segment was defined by its most caudal dorsal rootlets [15]. Every segment was placed at the center of one well of a 48-well plate. The well was then filled with 0.5 ml jellifying solution (sucrose 10% in phosphate buffer 1M; low melting point agarose [Sigma Chemical Co., St. Louis, MO] 4%). After 24 h storage at 4°C the jelly blocks were serially cut into 20 µm thick coronal sections using a vibratome (Leica VT 1000S, Germany). Sections were then mounted on jellified slides (unflavored gelatin 6 g; KCr(SO4)2.12 H2O 0.5 g, DW to 300 ml).

Sections were stained with the cresyl violet technique and used for cell counting and morphometric analysis. From each block, three to five slices, 120 µm apart, were analyzed. Additional sections were immunolabeled with appropriate antibodies.

### Bromodeoxyuridine injection protocol and tissue sections pretreatment

Additional five young and four aged female rats were given a single daily injection of 5-bromo-2′-deoxyuridine (BrdU; 50 mg/ kgi.p., Sigma, St. Louis, MO) during 10 days. Seventeen days after the last injection, animals were perfused and spinal cords processed as described above. For BrdU immunofluorescence different DNA denaturation variants using formamide in SSC were compared against the standard procedure described below. It was found that in all cases formamide caused a significant deterioration of section quality without a significant improvement of BrdU labeling intensity. Briefly, sections were hydrated with PBS during 10 min. Then, sections were treated with 2M HCl for 30 at RT. Samples where then washed with borate buffer 0.1 M,pH 8.5 during 10 min. Sections thus treated were used for immunofluorescence labeling.

### Immunofluorescence

For double-labeling BrdU immunohistochemistry, mouse anti-BrdU (DakoCytomation) and rabbit anti human neuron specific enolase (NSE; DAKO Corporation, Carpinteria, USA) were used as primary antibodies. Slides were washed twice with PBS, and incubated for 45 min with a 1∶1000 Alexa555-conjugated goat anti-mouse IgG and 1∶1000 Alexa488-conjugated goat anti-rabbit IgG (Jackson Immuno Research, West Grove, Pennsylvania). After washing the slides twice with PBS, they were counterstained for 15 min with the fluorescent DNA stain 4′,6-diamidino-2-phenylindole (DAPI). Fluorescence was detected with an Olympus confocal microscope (Olympus FV1000) with an emission filter of 490–540 nm, for Alexa488 detection (laser 473 nm); 575–675 nm for Alexa555 detection (laser 559 nm) and 430–455 nm for DAPI detection (laser 405 nm). An objective of 40X (UPlanSAPO) with a NA of 0.95 was used. With the performed staining BrdU was localized in the cell nucleus, whereas the cell-specific marker used was present in the cytoplasm.

### Image analysis

The images of cresyl violet stained spinal cord sections were captured using a digital RGB video camera (Olympus DP71, Japan) attached to a microscope (Olympus BX50, Japan). In order to create a complete map of the entire segment taken with a 40x objective, images were captured using a digital image analyzer (cellSens Dimension, V1.4, Olympus Corporation, Japan) and stitched using an automatic Multiple Image Alignment process. No further processing was necessary after obtaining the original images.

For counting and morphometric determinations the entire segment was analyzed. In order to determine the morphometric characteristics of neuronal bodies, segmentation based on color was performed [16]. Neurons were then characterized using the following parameters: cellular area (reports the area of each object) and mean diameter (reports the average length of diameters measured at 2 degree intervals and passing through the object's centroid – equidistant point to the borders in an irregular object). To manually and automatically differentiate neurons from glial cells slides were stained with cresyl violet since the dye does not label glial cell somas and stains differentially glial from neuronal nuclei [17]. Besides, glial nuclei diameter is below 5 µm while those of neurons are above that size.

Morphometric data were taken only from those neuronal somas that showed a delineated shape and a distinguishable nucleus. Similarly, only those cells that were recognized by the image analyzer, based on the staining or color pattern and on their size and shape, were included in the analysis. In addition, there was an independent observation by two morphologists, in order to eliminate glial cells from counting and morphometric characterization. When determining the mean area occupied by neurons in the gray matter, all the neuronal somas were considered.

For estimating the number of cells present in an entire cervical segment the following formula was applied [18]:
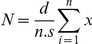



Where, N =  total estimated number of cellular bodies; d =  length (µm) of the rostrocaudal axis of the segment being assessed; n =  number of noncontiguous slices counted per cervical segment (n = 3); s =  thickness of the section (20 µm); x =  number of perikarya counted per non-contiguous slice assessed. Therefore, N represents an estimate of the total number of neurons present in every segment.

### Hormone assay

Serum PRL was measured by a specific radioimmunoassay using the rat materials provided by Dr. A. F. Parlow, Pituitary Hormones and Antisera Center, Harbor-UCLA Medical Center, Torrance CA, USA. Iodination grade rat PRL was radiolabeled by the Iodo-Gen® method [19]. A 1/10 goat anti-rabbit IgG serum in 0.05 M phosphate buffer, 1% normal rabbit serum and 8% polyethylenglycol, was used to separate bound from free hormone. Serum PRL was expressed in terms of NHPP rPRL RP-3.

### Statistical Analysis

To establish differences in the total number of neurons in the entire cervical region of young and aged rats the Student's t test for paired groups was applied. The analysis of variance (ANOVA) was used to evaluate differences between young and aged rats segments. Significant differences between mean values were defined as those with a p<0.05. The Correlation Coefficient was analyzed to determine correlation between the occupied neuronal area and the amount and size of neurons in young and aged rats.

## Results

### Age changes in the area of different cervical segments

**Figure 1:**
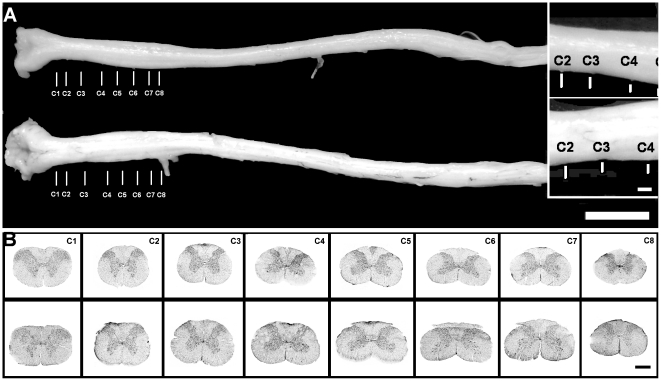
Low magnification view of the entire spinal cord of young and aged rats. A. The entire spinal cord was dissected from the spine both in young (above) and aged (below) rats after fixative perfusion. The vertical bars drawn below each spinal cord specimen correspond to the emergence of the nerve in each of the eight cervical segments. Note the increase in width of the entire aged spinal cord in comparison to the young specimen. Bar = 1 cm. Inset: magnification of the emergence of the C2, C3 and C4 nerves in the young (upper) and aged (lower) spinal cord to highlight the longitudinal enlargement of C3 in the aged specimen. Bar = 1 mm. B. Twenty µm thick coronal section of each cervical segment of young (upper panels) and aged (lower panels) female rats stained with cresyl violet are shown. Sections were morphometrically assessed and data statistically compared among both age groups. Bar = 1 mm.

**Figure 2:**
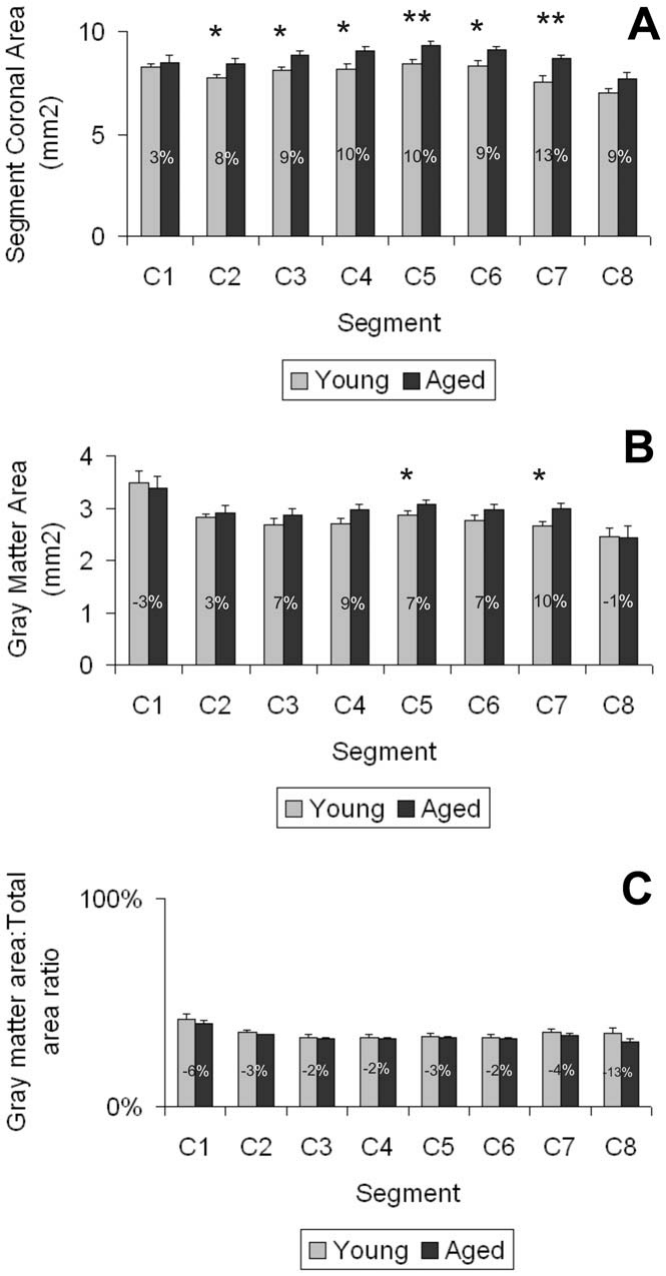
Age changes in the total and gray matter area in all cervical segments of female rats. The whole section (A) as well as the gray matter area (B) was manually delimited using an image analyzer and measured. White matter area was calculated by subtracting the gray matter area from the whole area of the section. Asterisks over bars indicate a significant difference (* P<0.05; ** P<0.001) from the corresponding young counterpart. The total cervical region area of aged animals was significantly greater than that of young rats. The gray matter area: total area ratio (C) was calculated in young and aged rats. Numbers on bars indicate the mean percentage of increase recorded.

Macroscopic and low magnification assessment of spinal cord sections corresponding to the cervical segments already revealed a statistically significant age-related increase in the overall area **(** **Fig. 1A and B** **)**. The absolute area (mm^2^) occupied by the gray and white matter in the entire cervical region showed a statistically significant increase with age. Although an increase in the absolute area was observed in all of the analyzed segments (up to 13%), statistical differences were detected only in C2 through C7 segments (**Fig. 2A**). The gray matter area increased in aged animals in comparison to young rats in all but C1 and C8 segments. Although differences with young rats were significant for the gray matter area of the whole cervical region, statistical differences between segments were only observed for C5 and C7 (**Fig 2B**). Despite the above changes, the ratio gray matter area:whole area did not change significantly with age (**Fig. 2C**).

### Morphometric analysis of age changes in cervical spinal cord neurons

**Figure 3:**
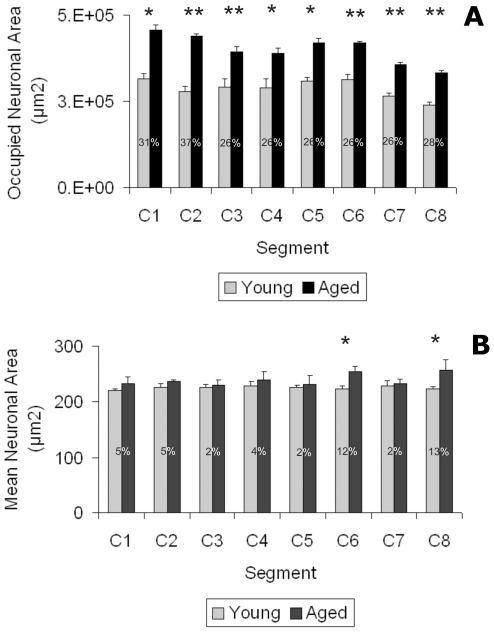
Morphometric analysis of age changes in cervical spinal cord neurons. The upper graph (A) shows the total occupied neuronal area in every cervical segment in young and aged rats. The lower graph (B) shows the mean neuronal area of the corresponding segment. Asterisks over bars indicate a significant (* P<0.05; ** P<0.001) difference from the corresponding aged counterpart. Numbers on bars indicate the mean percentage of increase recorded.

Aged rats showed a significant increase in the total area occupied by neurons in the corresponding segments **(** **Fig 3A** **)**. There was a trend towards an increase in neuronal size in the aged rats but this increase achieved significance only for C6 and C8 (**Fig 3B**).

**Figure 4:**
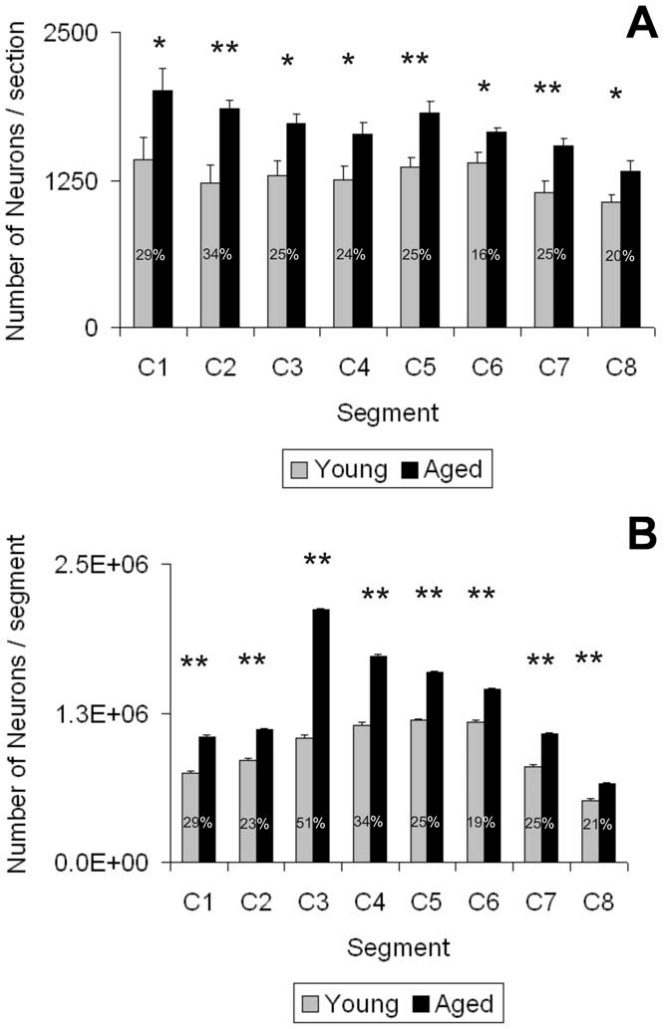
Cervical neuron numbers in young and aged rats. The upper graph (A) shows the average counting of neurons per section per segment of both age groups. The lower graph shows the estimated total number of neurons present in each segment. Asterisks over bars indicate a significant (* P<0.05; ** P<0.001) difference from the corresponding age counterpart. Numbers on bars indicate the mean percentage of increase recorded.

The average counting per section per segment (**Fig 4A**) as well as the estimation of the total number of neurons present in each segment revealed a neuron number increase ranging from 19% (C6) to 51% (C3) in the aged as compared to the young animals **(** **Fig 4B** **)**. A positive correlation was observed between the occupied neuronal area and the number of counted neurons per area both in young (r = 0.99) and aged rats (r = 0.95). On the other hand there was no correlation between the occupied neuronal area and the mean neuronal size (r = −0.22; r = −0.40, for young and aged rats, respectively).

### Immunofluorescence analysis in cervical spinal cells

**Figure 5:**
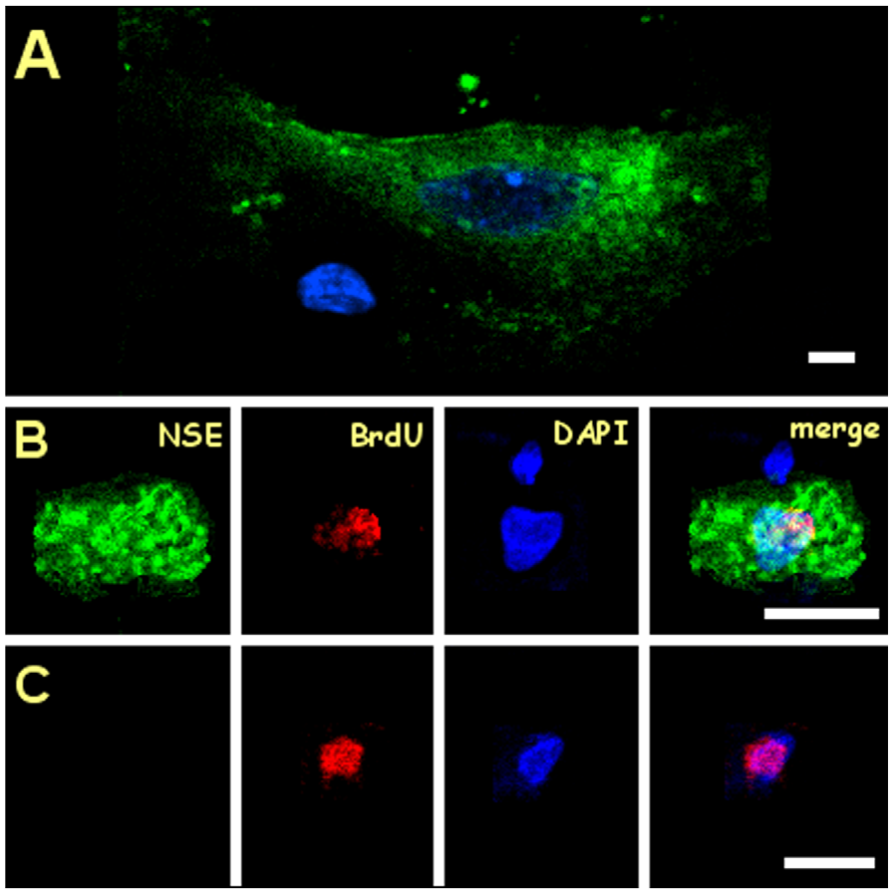
Presence of BrdU positive cells at the cervical spinal cord. **A.** A C6 young neuron labeled green (NSE) and blue (DAPI) negative for BrdU with a nearby negative glial cell. Bar = 20 µm. **B**. A NSE^[+]^ – BrdU^[+]^ neuron found in an aged C6 segment. Bar = 10 µm. **C**. A NSE^[−]^ – BrdU^[+]^ cells, probably corresponding to a glial cell, found in a young C4 segment. Bar = 10 µm.

Triple labeling immunofluorescence analysis showed the existence of both NSE^[+]^ - BrdU^[+]^ and NSE^[−]^ - BrdU^[+]^ cells in different segments of animals of both age groups **(** **Fig. 5** **).** In both groups, the frequency of BrdU^[+]^ cells in the cervical segments was very low. This fact made it difficult to statistically detect age-related differences of this parameter.

### PRL determination

As expected serum PRL levels increased with aging in the female rats **(** **Table 1** **)**.

**Table 1:** Cervical spinal cord neuron number and serum PRL levels in young and aged female rats.

Age	Total estimated neurons ^#^	Serum PRL (ng/ml)
Young	7×10^6^±0.12×10^6^ (n = 7)	24.0±1.9 (n = 6)
Aged	10×10^6^±0.08×10^6^ (n = 7)	110.0±4.6 (n = 5)
Significance	**	**

Data are expressed as mean ± SEM. Sections were stained with cresyl violet and neurons morphologically identified, classified by size and counted. #: Total estimated neurons in the entire cervical region. **: P<0.01

## Discussion

While previous studies indicate that in the cervical spinal cord of female rats, aging is associated with a variety of structural and cellular changes (see introduction), the present results reveal that the most significant changes take place in the gray matter where we found a significant increase in cervical neuron numbers in aged rats.

Comparison of the total and gray matter area of cervical segments between age groups provided the first indication of an increase in the number and/or size of gray matter elements with age. The H-shape of the gray matter has characteristic identity features for each cervical segment [20], which allowed us a reliable identification of all cervical segments in animals of both ages.

Since we previously showed that in the rat spinal cord the NeuN marker is lost with aging [9], we decided to identify neurons using a specific neuron cytoplasm marker (NSE) for immunofluorescence analysis. As was previously done [21], the cresyl violet staining was used for identification, counting and morphometric analysis of neuronal cells. This choice is based on the fact that the combination of size and morphology provides a safe set of criteria to perform reliable digital counting of spinal cord neurons. Considering that neurons are morphometrically well discernible from glial cells, in our opinion, this is a highly suitable technique for performing morphometry and counting.

Active cell proliferation has been reported in the spinal cord of juvenile turtles [22], young female mice [23] and young rats [3], but in neither case neurogenesis was detected. In the latter study, 4-month old male Fischer 344 rats were i.p. injected with BrdU and double labeling was subsequently performed in C7, T8 and L2 sections in order to detect cells showing colocalization of BrdU with different cell markers. Four weeks after BrdU injection, frequent cell proliferation throughout the spinal cord was observed, particularly in white matter tracts, but no BrdU-labeled cells were found to colocalize with markers of immature or mature neurons. Nevertheless, in a recent report the capability to initiate a neurogenic process in the gray mater of intact spinal cord has been shown in voluntarily exercising adult rats in a time-dependent manner [24]. Our results showing an increase of neuron numbers in all cervical segments of aged rats as well as the existence of cells positive for BrdU and NSE, strongly suggest the existence of neurogenesis in the spinal cord of adult and aged female rats. In search of an explanation that reconciles our results with those of Horner et al. [3], we considered the possibility that neurogenesis in the cervical spinal cord of female rats may be an event that occurs during discrete time windows.

Since we used females, the possibility exists that the physiological hyperprolactinemia that occurs during pregnancy and lactation gives rise to neurogenic waves in the spinal cord of pregnant and lactating mothers. This hypothesis is based on studies in pregnant female mice in which their physiological rise in circulating PRL levels was reported to induce neurogenesis in the forebrain subventricular zone [12], [13]. In these studies PRL was found not only to stimulate the proliferation of neuronal precursors but also to participate in the differentiation of these precursors into neurons.

In the female, but not male rat, aging is associated with a marked increase in the incidence of pituitary prolactinomas and mammary tumors [25], [26]. The prevalence of these pathological alterations begins to rise shortly after the first year of life [27]. These changes are paralleled by a sustained estrogen secretion, and low levels of circulating progestagens [28], [29]. It has been suggested that in female rats, continuous exposure to moderately increased or medium levels of estrogens unopposed by progesterone leads initially, to increased PRL secretion and later to the development of PRL-secreting pituitary adenomas [30]. Furthermore, it is known that an increased estrogen to progesterone ratio exerts an enhanced mitogenic action on a number of estrogen-responsive tissues. In this context, the progressive hyperprolactinemia and increased estrogen to progesterone ratio that develop in female rats during aging may directly or indirectly contribute to maintaining a positive rate of neuronal accumulation after reproductive age. Since aging is associated with a constellation of endocrine and other changes, a number of additional factors could also contribute to cervical neuron accumulation in very old animals.

It has been shown that progenitors isolated from the adult rat spinal cord in the presence of fibroblast growth factor, display stem cell properties and can generate neurons after transplantation in the adult rat dentate gyrus [31]. Consequently, it could be hypothesized that, when exposed to physiological cues *in vivo*, adult spinal cord progenitors possess the capability to differentiate into neurons.

Binucleated neurons have been described in the CNS of normal adult rabbits and rats [32]. They have been also found in substantial numbers in the cerebral cortex of the *flathead* mutant rat whose phenotype shows a marked reduction in the size of the cerebral cortex and cytokinesis failure in the developing pyramidal neurons [33]. Whether the presence of binucleated neurons in the C5 segments of our young and aged animals observed in previous studies [34] is related to the increase in neuron numbers with age is not clear at this stage.

Our results on the age changes of mean neuron size profiles in the cervical segments lend further support to the idea that in the gray matter of the cervical spinal cord, the neuronal populations are highly dynamic during the entire life span of the animals, with the larger neurons becoming predominant in the aged females. Our data also suggest that cytoplasmatic accumulation of lipofuscin pigments in aged neurons [7] while apparently devoid of toxic effects, may contribute to increasing cell size. As mentioned above, the expression of PTEN, a tumor suppressor gene known to play an important role in the regulation of cell size, has been shown to decrease in the spinal cord of aging rats [10]. This decrease may play a role in the age-related increase in neuronal size reported here.

It has been reported [7] that the mesenchymal cell marker vimentin showed a high level of expression in the basal cell layer surrounding the ependymal tube in young but not in aged females. Since the cell layer that surrounds or is close to the spinal cord central canal is believed to be a source of stem cells in the spinal cord [3], [35], the high expression of vimentin in the young rats may indicate a significant proliferative potential which seems to be substantially reduced in the aged animals. On the other hand, the expression of the glial markers S-100 and GFAP was comparable in young and aged animals with the latter being predominantly expressed in lamina X (surrounding the central canal). Since it is believed that GFAP labels stem cells [36], its periependymal distribution is consistent with the hypothesis that this region is rich in stem cells. The higher level of neurofilament protein expression in the cervical sections of aged versus young rats, in that study, is consistent with an increase in neuronal cell size and/or number. It is also consistent with the increase in the gray matter area and whole area observed in this and previous studies [7]. Interestingly, the possibility has been suggested that the ependyma of the rat spinal cord may be a reservoir of immature neurons in “standby” mode, with the potential to complete their maturation and integrate to spinal circuits [37]. The region that surrounds the central canal of the spinal cord derives from the neural tube and retains a substantial degree of plasticity. In turtles, this region is a neurogenic niche where newborn neurons coexist with precursors, a fact that may be related with the endogenous repair capabilities of low vertebrates. Immunohistochemical evidence suggests that the ependyma of the mammalian spinal cord may contain cells with similar properties, but their actual nature remains unsolved [37].

In functional terms, C5 is the cervical segment that innervates the largest number of muscles, including pectoral, thoracic, neck and forelimb (brachial plexus) muscular groups; C5, together with C3 and C4 motoneurons also contribute to diaphragm innervation [38]. Aging seems to have a differential impact on cervical and lumbar neurons. Thus, a significant decrease in the number of gastrocnemious, but no ulnar, motoneurons was reported in aged (27 mo.) versus middle aged (9 mo.) Fischer 344 males [39]. Also, sex seems to be an important determinant of the effect of aging on spinal cord neurons as indicated by the observation that very old (>30 mo.) WAGxBN male, but not female, rats undergo a high prevalence of paralysis or severe paresis of the hindlimbs and atrophy of the skeletal muscles in the lumbar region and hindlimbs [40]. In our rat colony, aged females (some of which live up to 33 months) virtually never show paralysis of the hindlimbs.

Although the evidence reported here is limited to a single region of the spinal cord and does not explore the influence of gender and other biological variables on spinal cord neurogenesis, a task beyond the scope of a single study, the importance of the present results lies in the fact that they provide two clear lines of evidence indicating that NSE-positive neuron number increase occurs in the adult and aged spinal cord of the female rat. Long term exposure to high levels of circulating PRL in the female rat may account, at least in part, for this phenomenon.

The present report extends the conclusions of previous studies in the brain of older humans (Eriksson et al., 1998) [1], in the sense that it suggests that the aging spinal cord of mammals also retains a significant degree of neuronal plasticity and could therefore be induced to undergo self-repair by proper activation of dormant physiologic mechanisms.

We conclude that in the female rat, aging is associated with an increase in the number and average size of cervical spinal cord neurons, thus increasing the overall cervical area volume. We suggest that specific endocrine changes that occur during the female rat life span such as rises in circulating PRL levels can trigger neurogenic processes responsible, at least in part, for the age-related increase in the number of cervical spinal cord neurons reported here. Whether neuron numbers increase with age in other spinal cord segments and whether this phenomenon also occurs in males, remains to be investigated.
